# Temperature Effects on Symptom Expression of Lettuce Black Root Rot Caused by *Berkeleyomyces rouxiae*

**DOI:** 10.1264/jsme2.ME25065

**Published:** 2026-01-16

**Authors:** Misaki Edamoto, Toshiyuki Usami

**Affiliations:** 1 Graduate School of Horticulture, Chiba University, 648 Matsudo, Matsudo city, Chiba 271–8510, Japan

**Keywords:** common bean, cotton, cowpea, okra, *Thielaviopsis basicola*

## Abstract

Lettuce black root rot caused by *Berkeleyomyces rouxiae* occurs during the hot season in Japan, whereas black root rot in other crops often develops during cooler seasons. The present study investigated the relationship between temperature and symptom severity in lettuce and other plant species. Inoculation tests conducted with different isolates revealed that symptoms on lettuce were the most severe at 25°C, whereas those on cotton, okra, and cowpea were the most severe at 15–20°C. These results align with the seasonal occurrence of lettuce black root rot in Japan. The present study provides valuable insights for predicting and managing this disease.

Black root rot, a soilborne disease caused by the fungal pathogen *Thielaviopsis basicola* (syn. *Chalara elegans*), has‍ ‍been reported in various plant species worldwide since the mid-1800s (Nel *et al.*, 2019). Infected plants exhibit necrotic lesions on root tissues, leading to suppressed growth. *T. basicola* was recently reclassified into the genus *Berkeleyomyces* and was divided into *Berkeleyomyces basicola* and *B. rouxiae* (Nel *et al.*, 2018). Black root rot‍ ‍of‍ ‍lettuce (*Lactuca sativa*) caused by *B. rouxiae* was first‍ ‍reported in Japan in 2016 (Nakane *et al.*, 2019). This disease, which was initially observed in lettuce grown in Gunma Prefecture, was subsequently detected in Nagano (Ishiyama, 2022), Shizuoka (Sasaki and Usami, 2020), and Ibaraki prefectures (Nakane and Usami, 2020). In all cases, the disease occurred in lettuce cultivated during the hot season. Consistent with this incidence pattern, Lloyd and Lockwood (1963) reported that the optimum temperature for pea black root rot caused by *T. basicola* was high at approximately 28°C. In contrast, black root rot in other plant species is often associated with cooler conditions. For example, Lloyd and Lockwood (1963) found that optimum temperatures for disease development on poinsettia and tobacco were 17–20°C and 17–23°C, respectively. Similarly, cotton black root rot was more prevalent at lower soil temperatures because of seasonal and geographical conditions (Mauk and Hine, 1988; Pereg, 2013). Rothrock (1992) also demonstrated that the incidence and severity of cotton black root rot were higher at 20–24°C. Additionally, black root rot of faba bean has been observed during the cool season in China (Long *et al.*, 2022).

Since lettuce black root rot is a recently recognized disease in Japan and because it has only been reported in a few other countries, such as Australia (O’Brien and Davis, 1994), the United States (Koike, 2008), and Brazil (Souza *et al.*, 2025), limited epidemiological information is currently available, particularly that related to the relationship between temperature and disease occurrence. Therefore, we investigated the optimum temperature for the symptom development of black root rot on lettuce and compared the results obtained with those for other plant species.

We used *B. rouxiae* isolates MAFF 247883 (from lettuce in Nagano Prefecture), MAFF 247972 (from lettuce in Shizuoka Prefecture), and MAFF 245175 (from pansy in Hokkaido Prefecture) (Oya and Usami, 2024, 2025). These isolates are available from the National Agriculture and Food Research Organization (NARO) Genebank (https://www.gene.affrc.go.jp/). Isolates were maintained on potato sucrose agar (PSA; potato broth prepared from fresh potatoes, 2% [w/v] sucrose, and 2% [w/v] agar) slants at room temperature.

To investigate the optimum temperature for mycelial growth, isolates were pre-cultured on PSA plates at 25°C for one week. After a mycelial disk with a diameter of 5‍ ‍mm was excised, it was placed in the center of a fresh PSA plate. Plates were incubated in the dark at 5, 10, 15, 20, 25, 30, and 35°C. Colony diameters were measured after the incubation, with five replicates per temperature treatment. All isolates grew at 10–30°C, with optimum growth being achieved at 25°C (Fig. 1). These results are consistent with previous findings for other *B. rouxiae* isolates (Nel *et al.*, 2018; Nakane *et al.*, 2019; Sasaki and Usami, 2020).

To investigate symptom severity at different temperatures, isolates were pre-cultured on PSA plates at 25°C for one week, transferred to potato sucrose broth (PSB; potato broth from fresh potatoes and 2% [w/v] sucrose), and incubated with shaking (120‍ ‍rpm) at 25°C in the dark for one week. Conidia were collected by centrifugation (1,500×*g*, room temperature) after filtering out mycelia with a single layer of KimWipe (Nippon Paper Crecia). They were resuspended in sterile distilled water at 10^6^ conidia mL^–1^. The roots of four-week-old seedlings of lettuce (cv. Summer Guy), okra (*Abelmoschus esculentus*, cv. Goryu), cotton (*Gossypium arboreum*), cowpea (*Vigna unguiculata*, cv. Juroku-sasage), and common bean (*Phaseolus vulgaris*, cv. Green Mild) were inoculated by root dipping in the conidial suspension. Seedlings were transplanted into plastic pots filled with vermiculite and supplied with diluted Hyponica nutrient solution (1:500; Kyowa) from the bottom. Plants were grown in chambers set at 15, 20, 25, or 30°C under a 12‍ ‍h light/12‍ ‍h dark photoperiod. One month after the inoculation, root rot symptoms were evaluated in the manner described by Oya and Usami (2025). Root discoloration severity was scored on a scale of 0–4: 0, no discoloration; 1, <25% roots discolored; 2, 25–50% discolored; 3, 50–75% discolored; 4, >75% discolored or complete root disintegration. The disease severity index was calculated as (Σ*SiPi*)⁄(4*Pt*)×100, where *Si* stands for the symptom score, *Pi* denotes the number of plants with that score, and *Pt* signifies the total number of plants. Disease severity indices were compared using the Steel–Dwass test in EZR v.1.68 (Kanda, 2013; Saitama Medical Center, Jichi Medical University; http://www.jichi.ac.jp/saitama-sct/SaitamaHP.files/statmedEN.html).

Representative results of inoculation tests using *B. rouxiae* isolate MAFF 247883 are presented in Fig. 2. Photographs of root rot symptoms are shown in Fig. 3. In lettuce, disease severity was significantly higher at 25°C (the Steel–Dwass test, *P*<0.05). In okra, severity peaked at 15°C. Although no‍ ‍clear optimum was found for cotton, common bean, or cowpea, symptom severity was generally higher at lower temperatures (15–20°C). Test results on plants inoculated with another isolate of the lettuce black root rot pathogen, MAFF 247972 (Fig. S1), were consistent with those obtained with MAFF 247883. The results of the inoculation with the pansy isolate, MAFF 245175, are presented in Fig. S2. Since this isolate is not pathogenic on lettuce (Oya and Usami, 2024, 2025), plant species other than lettuce were used for these tests. Although symptom severity on common bean at 15°C by the pansy isolate MAFF 245175 was lower than that caused by the lettuce isolates MAFF 247883 and MAFF 247972, the overall inoculation results on each plant species were nearly identical among isolates. Symptom expression at each temperature appeared to be independent of the fungal strain and its isolation source. Similar results were obtained from repeated inoculation tests conducted at least three times. A mock inoculation (roots dipped in sterile distilled water) led to no symptoms on roots.

The present results demonstrate that the optimum temperature for the symptom expression of lettuce black root rot is high (25°C). This result is consistent with the findings of field observations conducted in Japan, where lettuce black root rot primarily occurred during the hot season (Nakane *et al.*, 2019; Nakane and Usami, 2020; Sasaki and Usami, 2020; Ishiyama, 2022). In contrast, root rot symptoms in okra, cotton, cowpea, and common bean were more severe at cooler temperatures. Previous studies described similar patterns for cotton. Rothrock (1992) demonstrated that the‍ ‍symptoms of black root rot caused by *T. basicola* on *G.‍ ‍hirsutum* were the most severe at 20°C. Lloyd and Lockwood (1963) also reported 21°C as the optimum temperature for cotton. Similarly, Mauk and Hine (1988) found higher disease severity at 20°C than at 28°C, and higher infection rates at 18–20°C than at 25–27°C on *G. barbadense*. The present results, which were obtained using *G. arboreum*, suggest that high susceptibility to the disease at low temperatures around 20°C is a common feature among *Gossypium* species.

In lettuce, the optimum temperature for symptom expression (25°C) was consistent with the optimum for pathogen growth. However, this was not the case for other host plants. Lloyd and Lockwood (1963) also observed mismatches between optimum pathogen growth temperatures and disease severity in several hosts (*e.g.*, cotton, poinsettia, and pea). Similarly, Yan and Nelson (2020) reported that *Fusarium tricinctum* caused severe disease at 15°C despite its optimum growth temperature of 24.4°C. These findings indicate that symptom expression is not solely set by pathogen growth. Lloyd and Lockwood (1963) indicated that the disease severity of black root rot increased at temperatures that were less favorable for host growth. The results of our inoculation tests indicated that okra, common bean, cowpea, and cotton were highly susceptible to black root rot at temperatures that were unsuitable for their growth. A previous study reported that optimum temperatures for the growth of shoots and roots in okra, common beans, and cowpeas were 34, 28–30, and 32–34°C, respectively (Kadota, 1972). Reddy *et al.* (2017) showed that optimum thermal conditions (day/night temperature) for the growth of cotton (*G. hirsutum*) shoots and roots were 35/27°C. Conversely, temperatures <20°C were unsuitable for the growth of these plants (Kadota, 1972; Reddy *et al.*, 1997, 2017). Therefore, low-temperature stress may play a role in high susceptibility to root rot at 15–20°C.

On the other hand, the most severe root rot symptoms on lettuce occurred at 25°C. The vegetative growth of adult Empire type-crisphead lettuce plants, a similar type of lettuce to that used in the present study, is often suppressed at temperatures >25°C because this induces reproductive growth (Shibutani and Kinoshita, 1968). However, Noguchi *et al.* (1981) reported that the optimal growth temperature for this type of lettuce plant at the young stage (with 0–10 leaves) was 25°C. Therefore, a temperature of 25°C, at which the most severe symptoms were observed in the present study, does not appear to be a stressful temperature for lettuce. The optimal growth temperature for *B. rouxiae* was 25°C. Therefore, the severe symptoms observed on lettuce roots may be attributed to the pathogen’s high activity rather than by temperature stress on plants.

Symptom expression often involves complex interactions among pathogen traits, host physiology, and environmental factors. For example, although *F. oxysporum* f. sp. *lycopersici* and *F. oxysporum* f. sp. *radicis-lycopersici* both have optimum growth temperatures of 25–30°C, their optimum temperatures for symptom expression in tomato markedly differ at 25–30°C and 19°C, respectively (Clayton, 1923; Hibar *et al.*, 2006). This finding suggests that even with a similar pathogen physiology and host plant species, symptom expression may markedly vary depending on complex host–pathogen interactions. Since *B. rouxiae* infects multiple types of host plant species, the optimum temperature for disease development must be evaluated individually for each host.

Although many epidemiological aspects of black root rot remain unclear, particularly in non-lettuce hosts, the present study provides novel insights into the optimum temperature for symptom development in lettuce. These results will contribute to the more accurate prediction and management of lettuce black root rot.

## Citation

Edamoto, M., and Usami, T. (2026) Temperature Effects on Symptom Expression of Lettuce Black Root Rot Caused by *Berkeleyomyces rouxiae*. *Microbes Environ ***41**: ME25065.

https://doi.org/10.1264/jsme2.ME25065

## Supplementary Material

Supplementary Material

## Figures and Tables

**Fig. 1.**
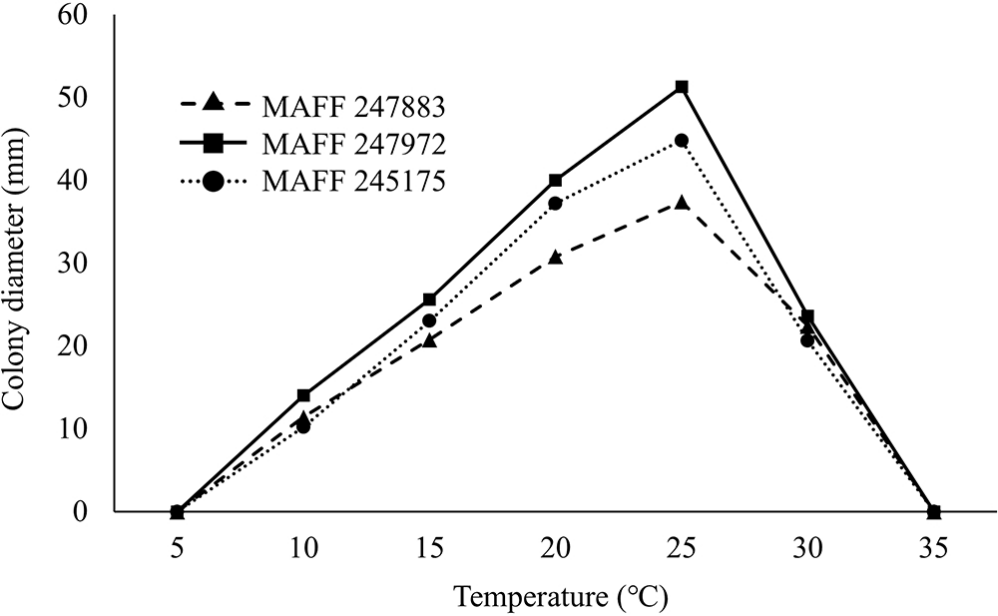
Mycelial growth of three *Berkeleyomyces rouxiae* isolates used for inoculation tests. Each isolate was cultured on PSA plates (five replicates per temperature) and incubated in the dark at 5, 10, 15, 20, 25, 30, and 35°C. Colony diameters were measured. Mean values were calculated.

**Fig. 2.**
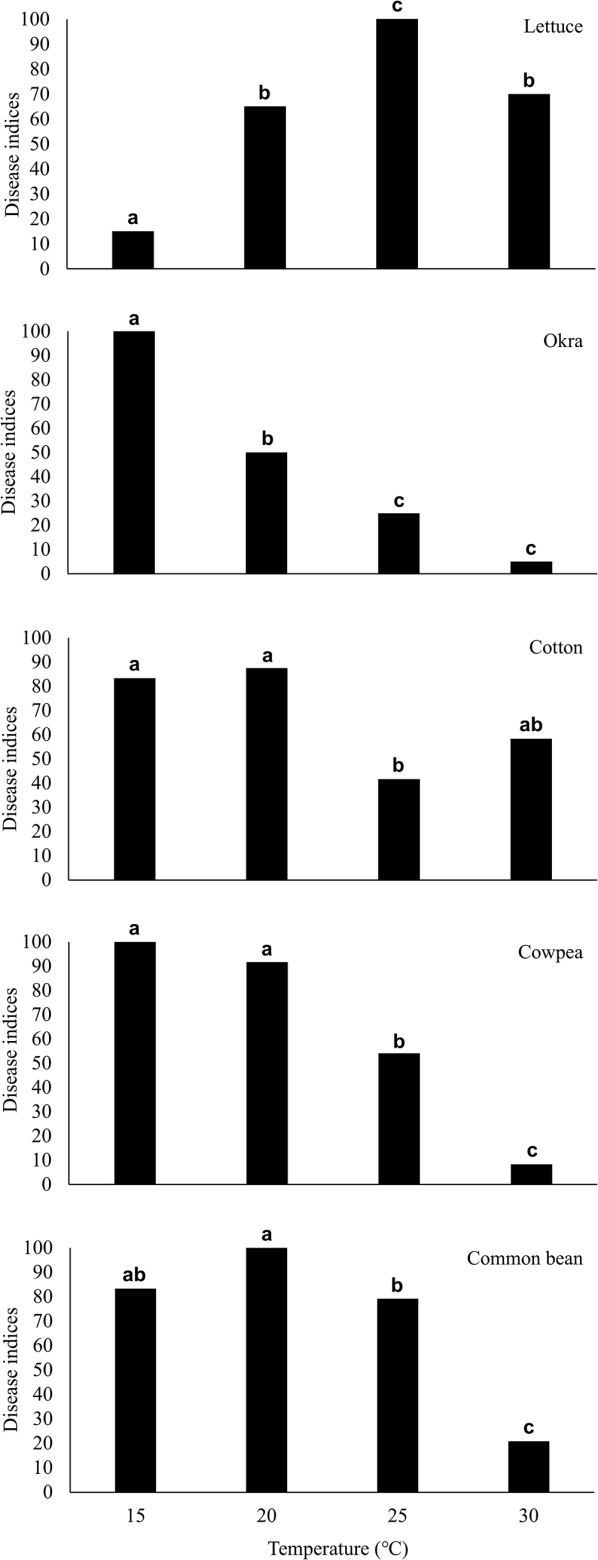
Disease index of black root rot on lettuce, okra, cotton, cowpea, and common bean inoculated with a lettuce isolate of *Berkeleyomyces rouxiae* MAFF 247883 at different temperatures. Different letters denote a significant difference in the disease indices found using the Steel–Dwass test (*P*<0.05).

**Fig. 3.**
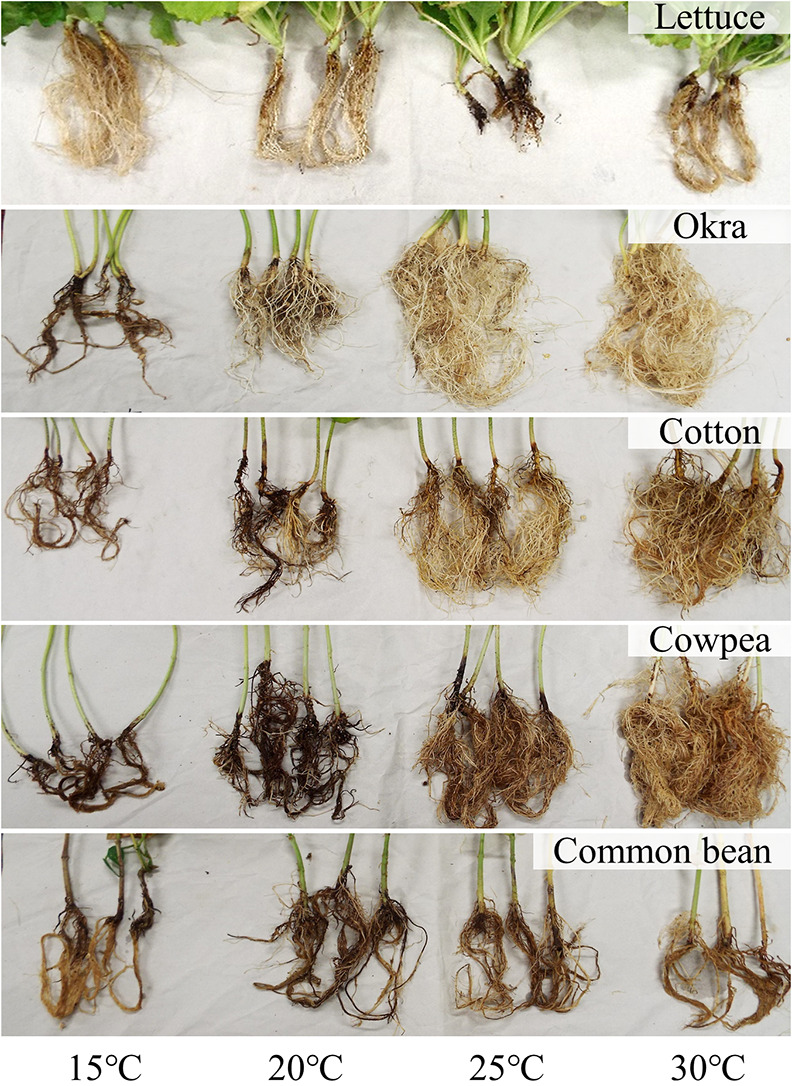
Pictures of root rot symptoms on lettuce, okra, cotton, cowpea, and common bean inoculated with a lettuce isolate of *Berkeleyomyces rouxiae* MAFF 247883 at different temperatures.
