# Supplementary material for: Temperature Effects on Symptom Expression of Lettuce Black Root Rot Caused by Berkeleyomyces rouxiae
Source: Microbes Environ. 2026 Jan 16;41(1):ME25065. doi: 10.1264/jsme2.ME25065 (PMC12999736; doi:10.1264/jsme2.ME25065)
Supplement: Supplementary file 1 — Supplementary Material [file 41_25065_s1.pdf]

Temperature effects on symptom expression of lettuce black root rot caused by *Berkeleyomyces rouxiae*

Microbes and Environments
Misaki Edamoto • Toshiyuki Usami*
Graduate School of Horticulture, Chiba University
*usami@faculty.chiba-u.jp

Supplemental figures

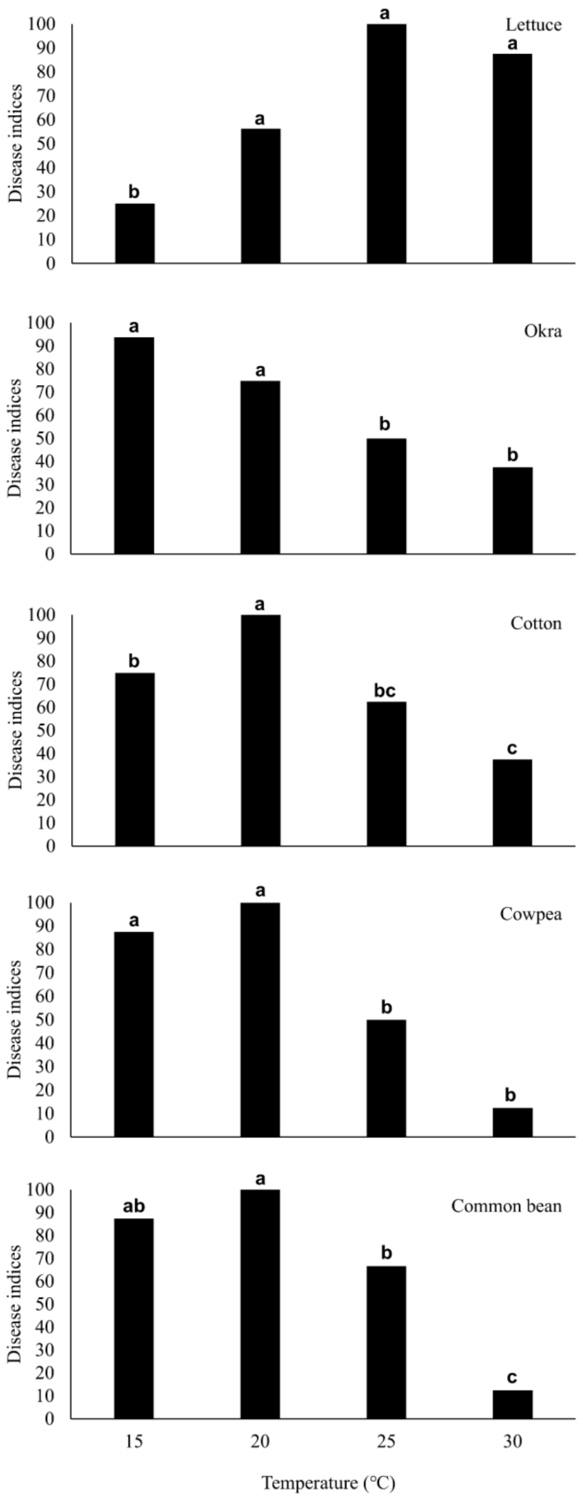

Fig. S1
Disease index of black root rot on lettuce, okra, cotton, cowpea, and common bean inoculated with a lettuce isolate of *Berkeleyomyces rouxiae* MAFF 247972 at different temperatures. Different letters denote significant difference in the disease index as found using Steel–Dwass tests ($P < 0.05$).

Temperature effects on symptom expression of lettuce black root rot caused by *Berkeleyomyces rouxiae*

Microbes and Environments
Misaki Edamoto • Toshiyuki Usami*
Graduate School of Horticulture, Chiba University
*usami@faculty.chiba-u.jp

Supplemental figures

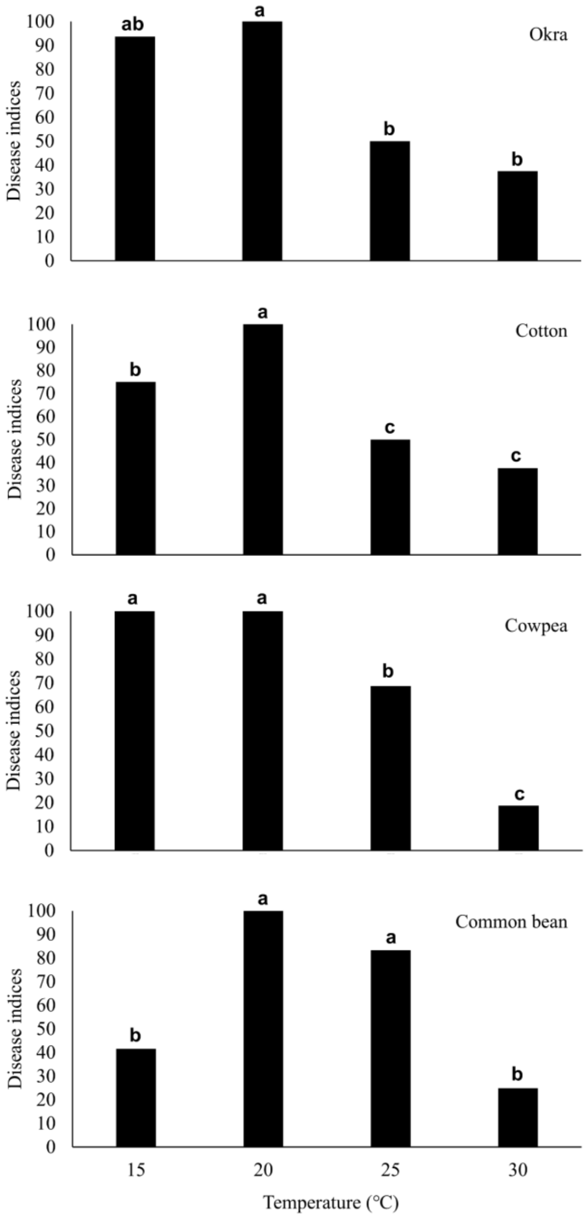

Fig. S2
Disease index of black root rot on okra, cotton, cowpea, and common bean inoculated with a pansy isolate of *Berkeleyomyces rouxiae* MAFF 245175 at different temperatures. Different letters denote a significant difference in the disease index as found using Steel–Dwass tests ($P < 0.05$).
